# How long does it take to complete and publish a systematic review of animal studies?

**DOI:** 10.1186/s12874-025-02672-5

**Published:** 2025-10-01

**Authors:** Julia Victoria Bugajska, Bernard Friedrich Hild, David Brüschweiler, Enrico Daniele Meier, Alexandra Bannach-Brown, Kimberley Elaine Wever, Benjamin Victor Ineichen

**Affiliations:** 1 Center for Reproducible Science, University of Zurich, Zurich, Switzerland; 2 QUEST Center for Responsible Research, Berlin Institute of Health at Charité- Universitätsmedizin Berlin, Berlin, Germany; 3 Department of Anesthesiology, Pain and Palliative Medicine, Radboud university medical center,; 4 Clinical Neuroscience Center, University of Zurich, Zurich, Switzerland; 5 Department of Clinical Research, University of Bern, Bern, Switzerland

**Keywords:** Systematic review, Animal research, PROSPERO, Pre-registration, Evidence synthesis, Meta-analysis, Animal welfare

## Abstract

**Introduction:**

Conducting a rigorous systematic review of animal studies requires a priori registration of a study protocol. However, it remains unknown how many of these registered studies culminate in publication and how long it takes to complete such a systematic review. Thus, this study had two objectives: (1) to assess the proportion of registered protocols that result in publication, and (2) to determine the time required to complete and publish systematic reviews of animal studies after protocol registration.

**Methods:**

All available systematic reviews protocols of animal study were manually downloaded from PROSPERO, the international registry of systematic review protocols. Start and completion date as well as topical and demographic data were extracted, complemented by a web-scraping approach. Assessment of publication status was achieved through a systematic literature search.

**Results:**

From a total of 1,771 protocols, 406 were excluded due to recent start dates. This left 1,365 protocols eligible for the final analysis. Among these, 694 (51%) resulted in a published systematic review. Median time to complete and publish a systematic review was 11.5 months (range: 0.13–44.9 months) and 16.2 months (range: 1.0-49.7 months), respectively. This time was 69% more until submission than anticipated by the authors (6.8 months [range: 0.9–48.0]).

**Conclusion:**

Only half of registered protocols resulted in publication, suggesting possible publication bias. Authors can expect to complete and publish an animal systematic review within approximately one year.

**Supplementary Information:**

The online version contains supplementary material available at 10.1186/s12874-025-02672-5.

## Introduction

By rigorously synthesizing data from various animal studies, systematic reviews provide a comprehensive overview of an evidence base [1]. Thus, systematic reviews of animal studies can uncover key gaps and problems in animal research, inform best practice guidelines, reduce research waste, enhance reproducibility, and inform translational research to benefit human health [2–8]. This practice not only enhances the quality and credibility of research but also ensures ethical considerations in the use of animals [9].

A key step in a rigorous systematic review is the preregistration of a study protocol [10, 11]. This protocol outlines the review’s purpose, hypothesis, and methods and is usually registered on online platforms such as PROSPERO, which contains a section dedicated to protocols of systematic reviews of animal studies (PROSPERO4animals) [12], and accepting systematic review protocols for animal studies since 2018. For systematic reviews of clinical studies, it has been shown that a substantial part of these protocols do not result in publication [13, 14]. However, it is unclear if the same applies to animal research systematic review protocols. A quantitative assessment of this is needed to evaluate the extent of publication bias—the selective publication of studies based on their results [15]—while acknowledging that other factors, such as time constraints, resource limitations, or methodological challenges, may also contribute to non-publication. The lack of publication distorts the evidence base and can lead to misleading conclusions in research synthesis [16]. In addition to that, researchers often wonder about the time it takes to complete and publish an animal systematic review [17, 18]. Providing an empirical estimate of this timeframe could encourage more researchers to use systematic review and help streamline future reviews. Furthermore, understanding which countries and topics are represented in these protocols is relevant for identifying gaps where targeted educational initiatives could improve review quality, and for exploring whether cross-country collaborations influence the likelihood of publication.

Therefore, our study aims to: (1) Determine how many registered protocols on PROSPERO4animals result in published systematic reviews, including the countries involved and the topics covered, to identify potential targets for education and implementation of findings; and (2) Establish the timeline from registration on PROSPERO to when the systematic review is completed and published.

## Methods

### Study design

Methodological umbrella review of systematic review protocols for animal research registered in PROSPERO.

### Study registration

The protocol for our study was registered on the Open Science Framework (OSF) platform on March 30, 2024 (https://osf.io/up5zr/). We used the OSF protocol template for our study protocol.

### Search strategy and data extraction of PROSPERO protocols

We downloaded all animal systematic reviews protocols (i.e., falling under the category “Reviews of animal studies for human health”) from PROSPERO4animals on 31 January 2023 (*n* = 1,771). To account for the delays between anticipated/actual start date and publication, we excluded protocols with a start date within 466 days prior to data extraction. This cutoff was chosen post hoc, based on our analysis of the median time from protocol start to publication among all identified systematic reviews (see Results). From these protocols, we manually extracted the following data: subject index terms, contact details of authors, risk of bias and/or quality assessment tool(s) used, countries in which the review was conducted, the anticipated start date the anticipated completion date, the date of first submission, and the date of registration of the first version of the protocol. The protocol topics were manually classified into disease categories according to the International Classification of Diseases (ICD) 11th Revision.

We automated the process of data extraction for subject index terms, risk of bias and/or quality assessment tool(s) used, countries, anticipated start and completion date, and date of first submission to PROSPERO and registration of the first version by a web-scraping approach developed in Python version 3.10.12. The following packages were used: gspread version 5.11.1, oauth2client version 4.1.3, selenium version 4.12.0, time (Python standard library), bs4 (BeautifulSoup) version 0.0.1, re (Python standard library). All code to reproduce the data extraction of this paper are available on OSF: https://osf.io/up5zr/.

For the final analysis, we excluded protocols which had incomplete metadata in at least one of the following categories: anticipated or actual start date, submission date to journal, publication date in journal, date of registration in PROSPERO, date of submission to PROSPERO, subject index terms, or unclear publication status.

### Assessment of publication status

To determine if a protocol led to a published systematic review, we primarily searched the PROSPERO database for animal systematic review protocols reporting details of the final publication in field 40 “Details of final report/publication(s) or preprints if available”. We complemented this analysis by conducting a literature search using Google Scholar, PubMed, and Embase. We included Google Scholar in our search strategy due to its comprehensive coverage and sensitive search capabilities, including full-text searching and also covering preprints. This feature positions Google Scholar to potentially identify relevant studies that might be overlooked by the search functions of the other two commonly used biomedical databases, PubMed and Embase.

**Fig. 1:**
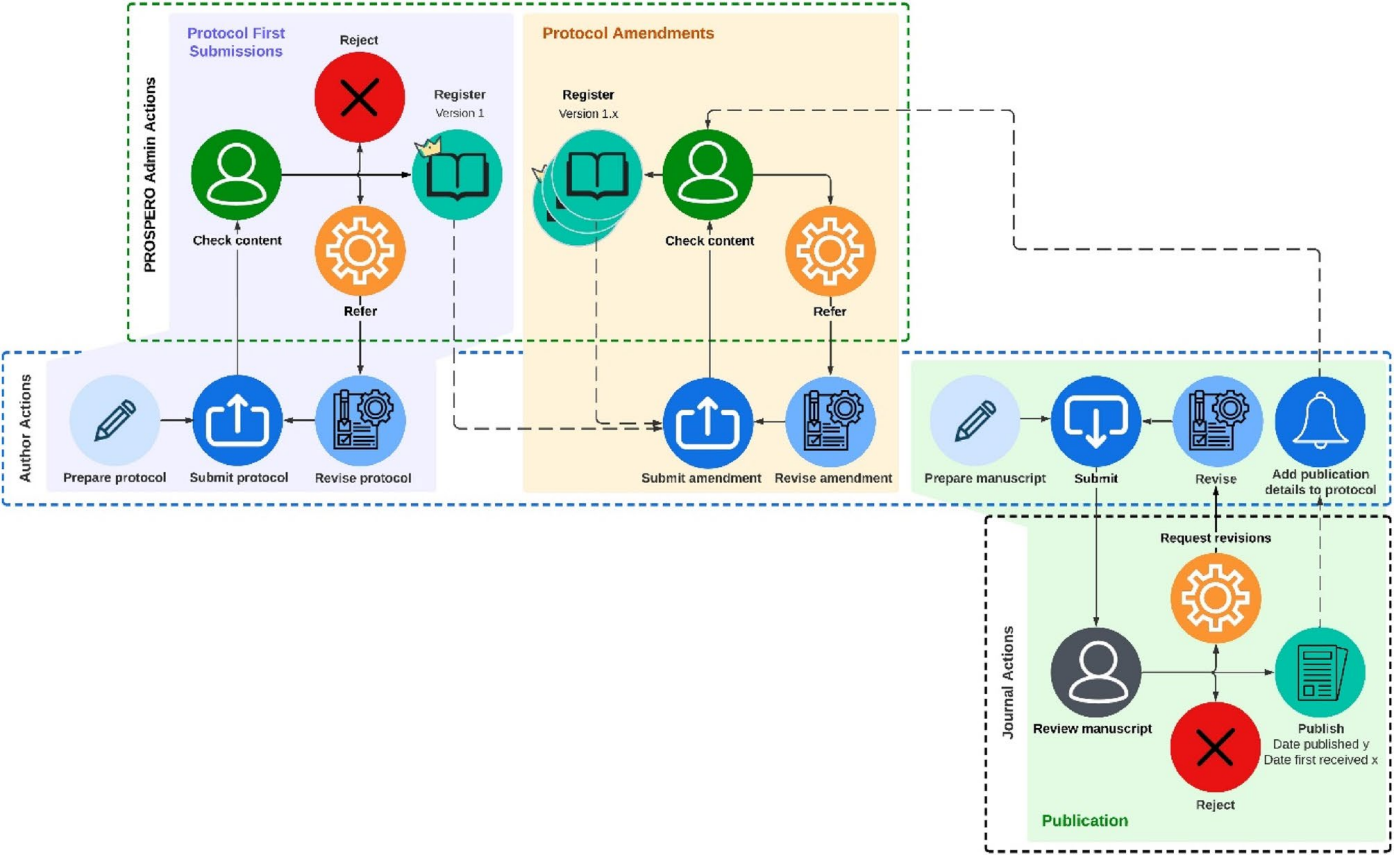
PROSPERO4animals systematic review protocol registration, amendment, and publication process

The initial step was to search for the registration number of the protocol on PROSPERO. If this did not yield results, we used Google Scholar, PubMed, and Embase to search for the title of the protocol (verbatim). Note that Google Scholar uses a flexible search syntax, which typically retrieves publications even when the titles differ between the protocol and the final publication. During this search, we compared the topic, title, and author names of any identified publications or preprint with those of the original protocol to confirm a match. In case a protocol did result in a published systematic review, we collected specific information from the publication, including journal name, the dates when the study was submitted and published, and the digital object identifiers (DOIs). If these methods were unsuccessful in identifying a published systematic review, we concluded that no such review had been published. We equaled the submission of a systematic review to a journal as the completion of the review. The processes relevant for protocol registration, amendments, and publication of respective systematic reviews are depicted in Fig. 1.

### Data synthesis and analysis

We conducted all statistical analyses in the R programming environment (version 4.2.2) [19]. We calculated descriptive statistics for the percentage of protocols resulting in published systematic reviews. Moreover, we calculated the time from anticipated starting to anticipated completion date as well as the actual time from starting time to paper submission/publication. We also assessed the most prolific countries on published protocols and percentage of protocols resulting in published papers. Finally, as post-hoc analysis, we evaluated whether the involvement of at least 4 authors or cross-country collaborations (≥ 2 countries involved) contributed to extended durations for completing the systematic review using Wilcoxon signed rank tests. For all data, we are reporting medians and interquartile ranges (IQR) based on normality testing using Kolmogorov–Smirnov and Shapiro–Wilk tests. A two-tailed P value < 0.05 was considered statistically significant.

### Data and code availability

The datasets including the analysis code supporting the conclusions of this article are available in the Open Science Framework repository, at https://osf.io/up5zr/.

### Deviations from the protocol

There were four notable deviations from our original study protocol:

First, we did not specify in the protocol exclusion of recently registered reviews. However, we excluded protocols with a start date within 466 days prior to data extraction, as it was unlikely these had already reached publication stage. This cutoff was chosen post hoc, based on our analysis of the median time from registration to publication (see results section), and was done to ensure sufficient follow-up time and to avoid underestimating time to publication. Including very recent protocols would bias results downward, as these reviews are likely ongoing but would be misclassified as unpublished. We applied the cutoff to both the publication proportion and time-to-publication analyses.

Second, although the protocol planned for contacting study authors to verify publication status, we did not conduct this step. This decision was due to time constraints, as the study was conducted as part of a master’s thesis, and based on prior experience showing low response rates in similar studies.

Third, we did not assess concordance between registered protocols and published reviews in terms of risk of bias tools, abstract screening procedures, or data extraction methods. This analysis is ongoing but was excluded here to avoid overloading the present study with information.

Fourth, we added an exploratory analysis to assess whether the number of authors or international collaboration influenced the likelihood of publication. This was not pre-specified but was included to explore potentially relevant factors.

## Results

### Research question 1: how many registered protocols result in published systematic reviews, and what countries and topics are involved?

#### Eligible protocols, proportion of published protocols, and number of authors involved

**Fig. 2:**
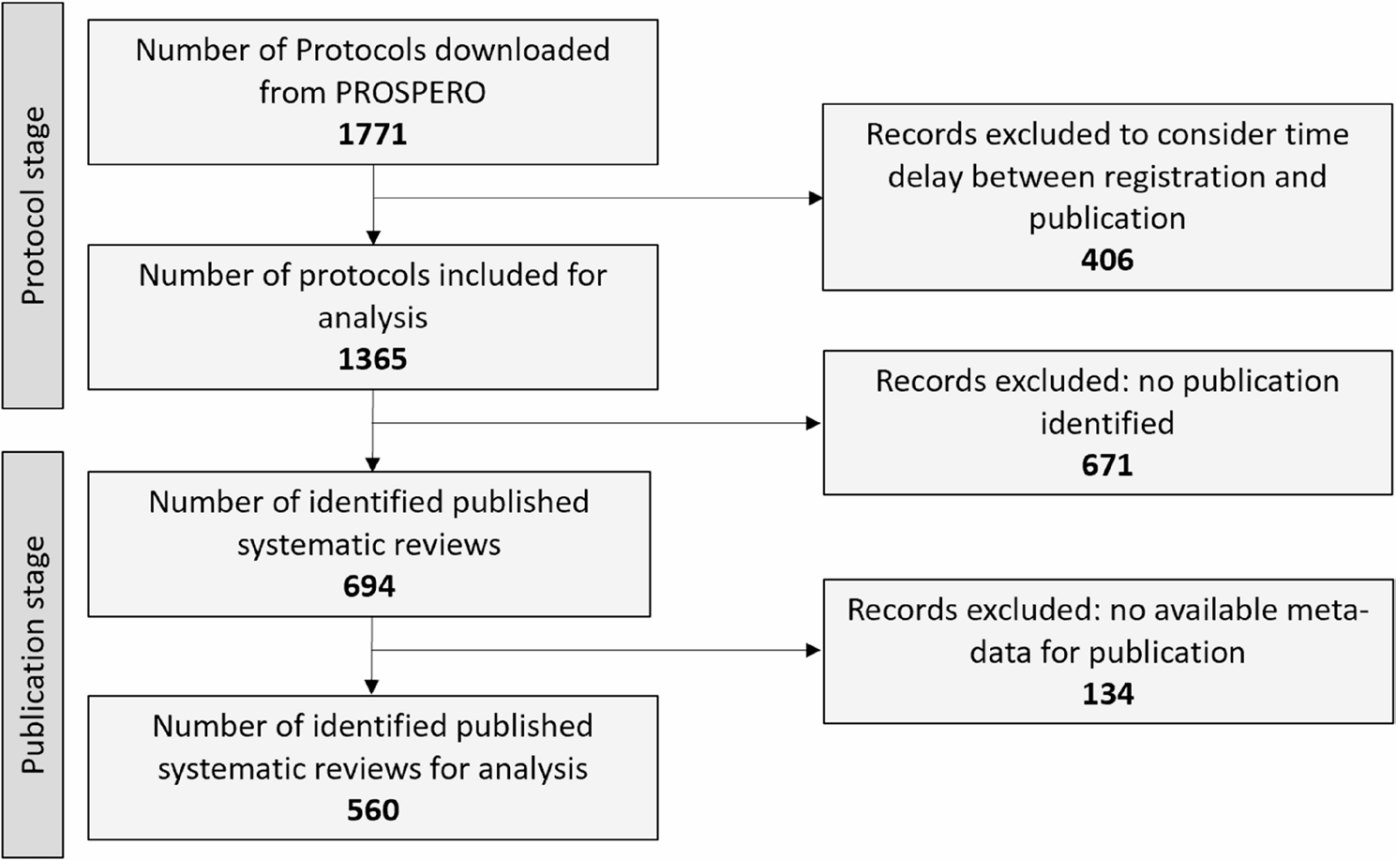
Study flow chart

We identified 1,771 protocols in total, of which 765 (43%) resulted in a published systematic review (Fig. 2, see Supplementary Data or https://osf.io/up5zr/ for the full dataset). To account for the time lag between registration and completion, we excluded protocols with a start date within 466 days prior to our final data extraction, as these reviews were likely still ongoing. This resulted in a final sample of 1,365 protocols, of which 694 (51%) led to publication. Of these, 120 (17%) had been marked as published by the authors directly on PROSPERO. Among these published reviews, 560 provided complete data including submission and publication dates in journals. All following analyses are based on this set of 560 records.

The identified systematic reviews were published across 330 different journals. We observed a wide distribution of the publications among these journals. The five most prevalent journals were “Frontiers in Pharmacology” (16 publications), “Neuroscience and Biobehavioral Reviews” (16 publications), “PLOS One” (16 publications), “International Journal of Molecular Sciences” (13 publications), and “Nutrients” (11 publications), jointly accounting for 13% of all publications.

The median time from journal submission to publication, i.e., the peer-review process, was 119.5 days, varying widely from 12 to 753 days. Detailed timelines for each journal releasing at least three publications can be found in Supplementary Table 1.

The median number of authors listed on the systematic review publications was 4 authors, with a range from 1 to 16 authors.

We identified eight protocols that were clearly registered after submission to a journal or after publication, constituting retrospective registrations.

#### Topics and research fields covered by the systematic review protocols

**Fig. 3:**
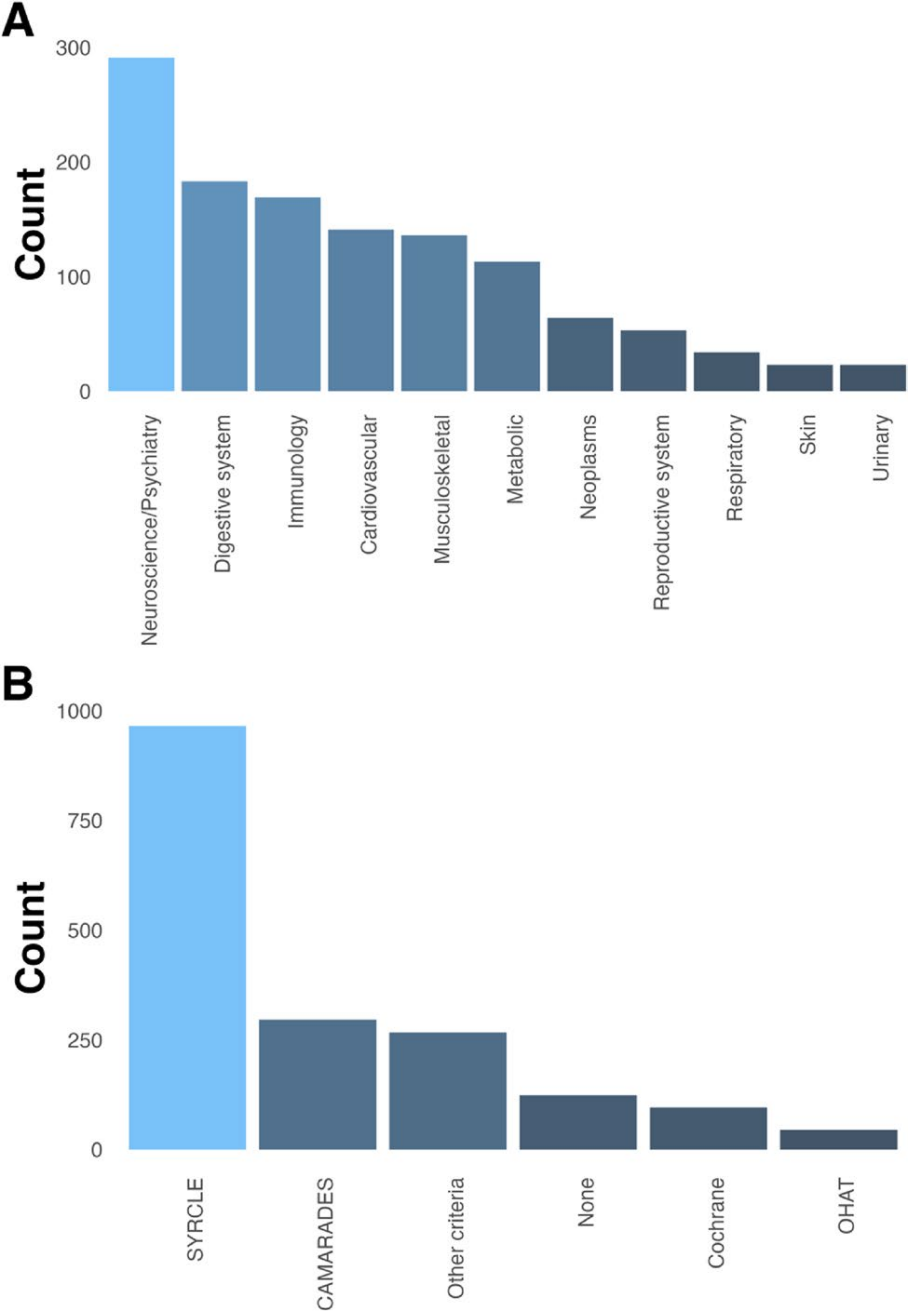
Covered research fields and risk of bias assessment tools reported in all protocols. **A** Incidence of disease areas mentioned in the registered protocols, classified according to ICD-11 code. Neuroscience/psychiatry was the most frequent topic. **B **Incidence of the risk of bias or quality assessment tools mentioned in the registered protocols. SYRCLE = SYRCLE’s risk of bias tool [20], CAMARADES = CAMARADES quality checklist [21], Cochrane = Cochrane’s risk of bias tool, OHAT = OHAT Risk of Bias Rating Tool for Human and Animal Studies. Abbreviations: OHAT, Office of Health Assessment and Translation

The most frequent research fields covered by the systematic review protocols were neuroscience/psychiatry (291 protocols, 21%), followed by digestive/dental science (183 protocols, 13%), and immunology (169 protocols, 12%) (Fig. 3A. See Supplementary Table 2 for more detailed numbers per subfield). 147 protocols (11%) intersected across multiple biomedical research domains. 281 protocols (21%) were not assigned to any research field.

#### Employed risk of bias and quality assessment tools

Most protocols reported using the SYstematic Review Centre for Laboratory Experimentation (SYRCLE) risk of bias tool (*n* = 965, 70.6%), followed by the Collaborative Approach to Meta-Analysis and Review of Animal Data from Experimental Studies (CAMARADES) checklist (*n* = 296, 22%). Other risk of bias or quality assessment tools were less commonly reported (*n* = 267, 20%) (Fig. 3B). 392 protocols (29%) reported that they planned to use more than one risk of bias or quality assessment tool.

#### Productivity of protocol registration by country

**Fig. 4:**
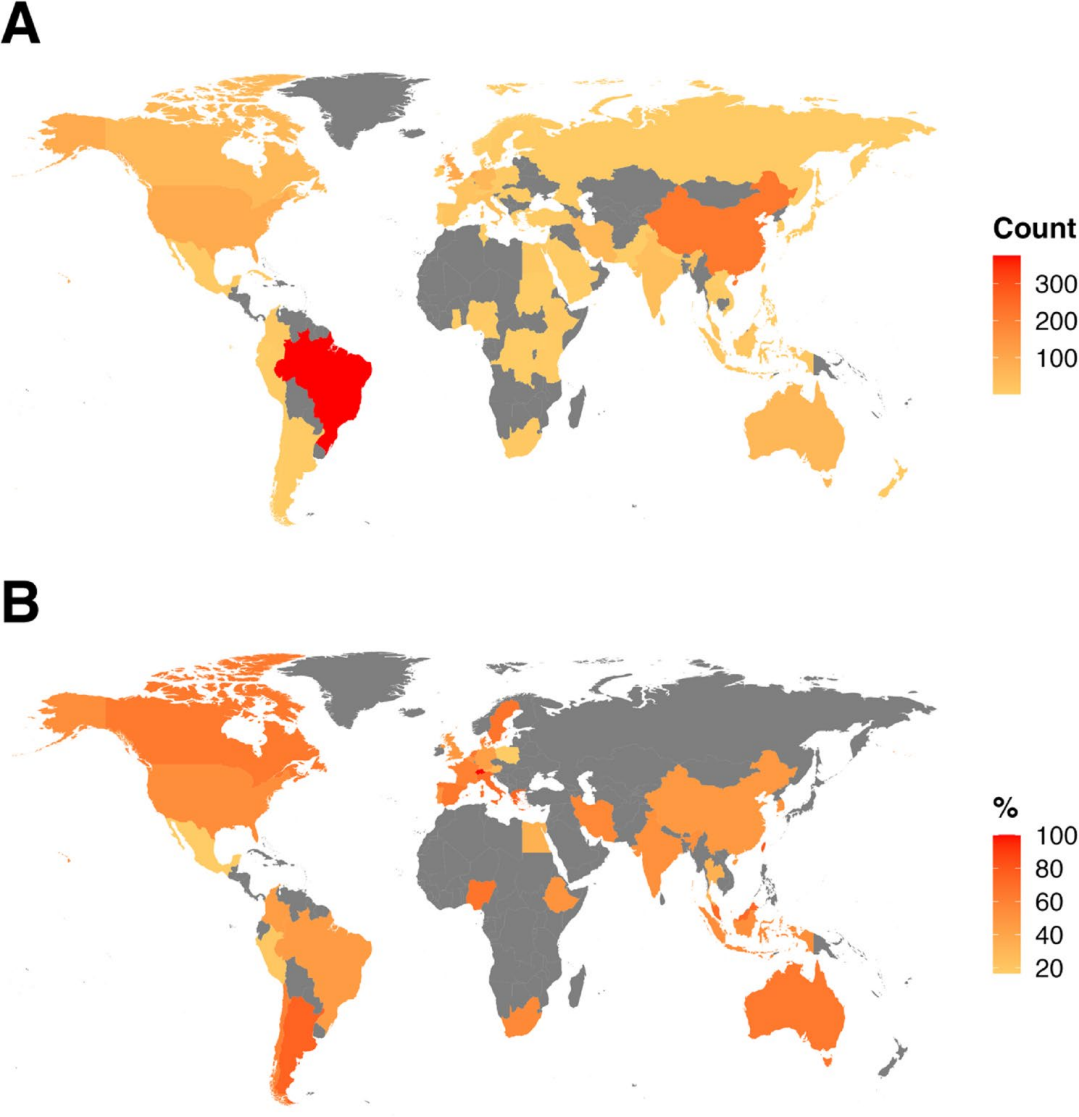
Global heat map of registered protocols and protocols resulting in a published systematic review. **A** Heat map of the absolute number of protocol registrations per country. **B** Heat map of the publication rate of registered protocols per country (only considering countries with ≥ 4 registered protocols)

The median number of systematic review protocols registered by each country was four (range 1 to 376). The top five prolific countries in terms of the number of registered protocols were Brazil (376 protocols, 28% of total protocols), China (216, 16%), the UK (94, 7%), The Netherlands (93, 7%), and the USA (92, 7%) (Fig. 4A). These countries were also the leaders in terms of the absolute number of protocols resulting in published systematic reviews, though the order differed: Brazil (171 publications, 45% of all Brazilian protocols), China (102, 47%), the Netherlands (60, 64%), the USA (49, 53%), and the UK (45, 48%) (Fig. 4B).

When limiting the analysis to countries with at least 10 registered protocols, the highest publication rates were observed for protocols from Malaysia (72%, 25 protocols/18 publications), Italy (70%, 37 protocols/26 publications), Spain (67%, 24 protocols/16 publications), Netherlands (65%, 93 protocols/60 publications), and Australia (64%, 56 protocols/36 publications).

### Research question 2: what is the timeline from protocol registration to completion and publication of systematic reviews?

**Fig. 5:**
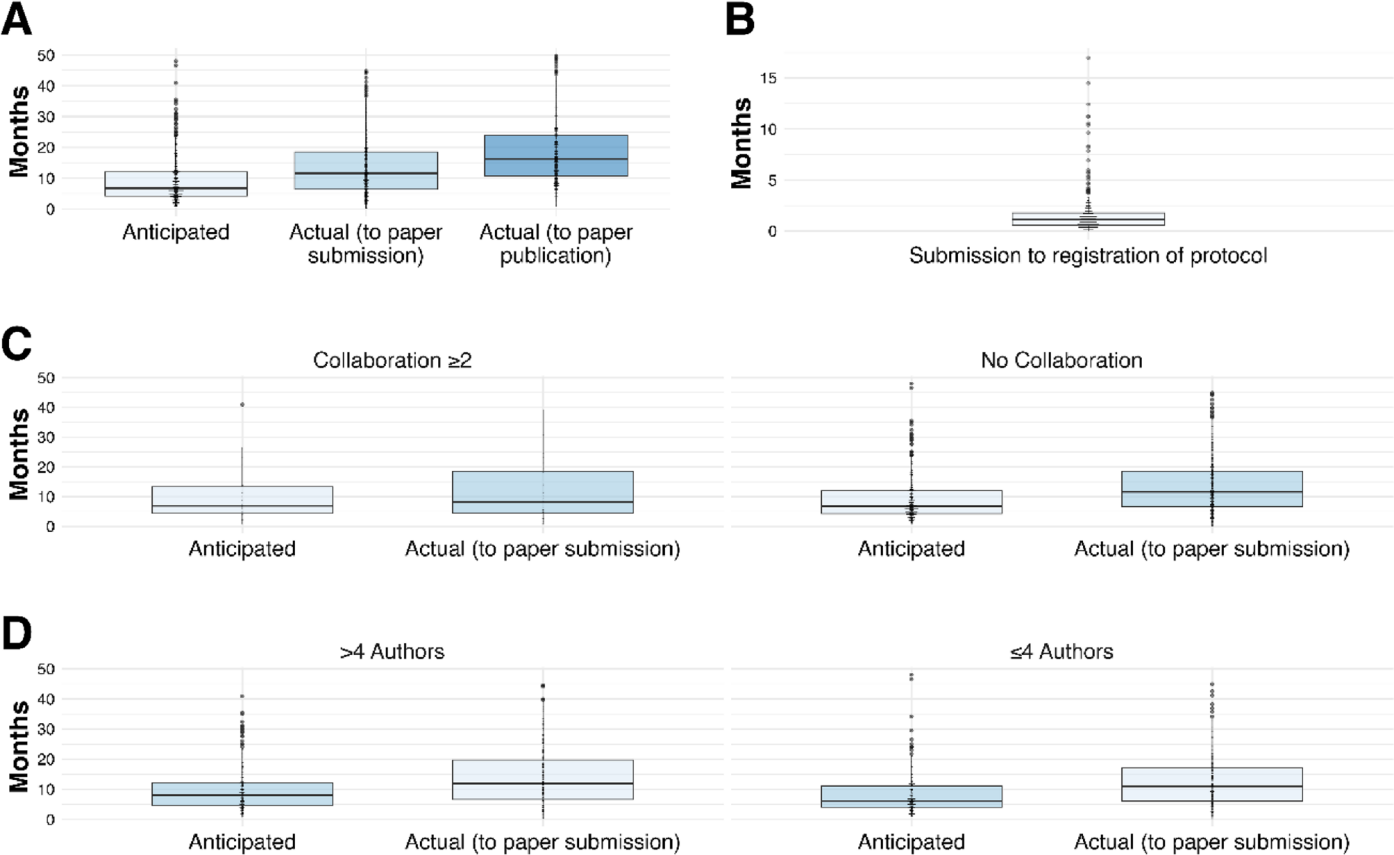
Timelines for protocol registration, systematic review completion and publication. **A** Times for anticipated completion (by the authors), actual completion, and publication. **B** Times from protocol submission to registration in PROSPERO. **C** Comparing anticipated and publication times for systematic reviews involving > 1 or only 1 country. Having cross-country collaboration does not significantly increase time to publication. **D** Comparing anticipated and publication times for systematic reviews involving > 4 or ≤ 4 authors. Having a larger author team does not significantly increase time to publication

To complete their review, authors estimated a median duration of 6.8 months (range 0.9–48.0 months) from the (anticipated) start date of their review to the anticipated date of completion (Fig. 5A). In contrast, the actual median time required to complete the review (i.e., time until submission to a journal) was 11.5 months (range 0.13–44.9 months). In addition, the median time required from the (anticipated) start date to manuscript publication was 16.2 months (range 1.0-49.7 months), respectively. Thus, the actual time needed to complete the systematic reviews were 69% longer than anticipated until submission. From the date of their first submission, protocols spent a median of 1.1 months (range 0–17 months) undergoing review by the PROSPERO4animal administrators and revisions by the authors before being registered (Fig. 5B).

Neither involving authors from more than 1 country nor involving more than 4 authors significantly prolonged the time to complete the systematic review (11.6 months [range: 0.13–44.8] versus 8.3 months [0.85–39.2], *p* = 0.51, and 11 months [range: 0.1–44.9] versus 12 months [0.1–44.5], = 0.70, respectively [Wilcoxon signed rank tests]) (Fig. 5C and D).

Of note, 8 of the protocols we included were registered on PROSPERO4animals after they had already been submitted to the peer-reviewed journals where they were eventually published (i.e., the publication date preceded the protocol registration date, indicating retrospective registration).

## Discussion

### Main findings

This study aimed to determine the proportion of registered study protocols that successfully led to published animal research systematic reviews. We also sought to assess the typical duration required to complete and publish these reviews. We show that around half of the systematic review protocols actually reach the publication stage. It takes a median of about 11 months to complete a review. This timeline appeared similar regardless of team size or international collaboration.

### Findings in the context of existing evidence

In our analysis, only about half of the initiated reviews reached the publication stage, alluding to publication bias of these systematic reviews. This is contrasted with observations from systematic reviews of human clinical studies: For instance, one study employing a web-scraping approach from the PROSPERO database found only 5% of clinical review protocols led to publication [22]. Yet, another study reported a much higher rate of 74% for protocols registered in PROSPERO’s first year [23]. The reasons behind the incomplete or unpublished status of systematic reviews may differ from those affecting primary research and can include unforeseen changes in the author team, diminishing interest in the topic, difficulties in finding a publishing journal, lack of a concomitant meta-analysis, or a combination of these issues [24]. A recent study highlights that systematic reviews conducted in developed, English-speaking countries, and those with external funding, have higher chances of publication [24].

The median time to complete an animal research systematic review was 11.5 months. However, the time span varies widely, ranging from less than a month to over 44 months. The median time to publish an animal systematic review was 16.2 months. This is slightly shorter than publishing a systematic review of human clinical data, which is estimated to take a median of 17 months to complete [17]. Of note, our data suggest that authors tend to underestimate time requirements with the actual review taking around 2/3 longer than anticipated. Additionally, our data highlights a delay, approximately five months, between the completion of a systematic review and its publication. While such delays are common in scientific publishing [25], they are particularly problematic for systematic reviews due to their time-sensitive nature [26]; the longer the publication process, the greater the risk of the review becoming outdated. To mitigate this issue, we recommend promptly making the completed systematic reviews accessible [27], for example, by utilizing preprint platforms like *MedArx*, *BioArx*, or *MetaArx*. This approach can ensure timely dissemination of findings. Another recently proposed method is the use of ‘living evidence summaries’ which are continuously updated to reflect the latest data and findings in the field [28]. Further research is needed to establish whether there is a risk of time-lag bias or publication bias—i.e., whether the likelihood or timing of publication depends on the review’s results. For example, inconclusive or null findings may be perceived as less publishable, which could delay submission or result in non-publication. This pattern has been observed in clinical trials, where studies with statistically significant or “positive” results are more likely to be published and published more quickly than those with non-significant findings [29, 30]. Similar mechanisms may plausibly affect the publication of systematic reviews. This highlights the importance of preprint platforms and living evidence summaries to support the timely dissemination of findings regardless of their conclusiveness.

We identified a small number of systematic reviews conducted by only one author. Such reviews raise concerns about methodological quality, as key steps—such as dual abstract screening, data extraction, and risk of bias assessment—should involve at least two independent reviewers to minimise biases and errors. In addition, it is unlikely that one person alone possesses the full range of expertise required for high-quality systematic reviews, including topic knowledge, systematic review methodology, search strategy design, and critical appraisal.

Our data indicate that neuroscience is one of the most frequently investigated fields, consistent with its prominence in both primary research and evidence synthesis globally [31, 32]. Several countries—particularly the USA, UK, the Netherlands, China, and Brazil—submitted a high number of protocols and showed similar output in terms of resulting publications. Three of these countries host branches of CAMARADES, a global initiative supporting the rigorous planning and conduct of systematic reviews of animal studies [1]. The Netherlands also hosted SYRCLE, which has played a key role in promoting methodological standards for such reviews [33].

Our study suggests a key area where educational initiatives could improve the rigour of animal systematic reviews. Concretely, we found instances where systematic reviews were submitted to journals before their protocols were officially registered. This suggests that the authors presented the process stage of their review incorrectly at the time of registration and that, in fact, their protocols would not have been eligible for registration on PROSPERO. In addition, many authors failed to update PROSPERO once the review was published, limiting the reliability of registry-based tracking. This highlights the need for more comprehensive education for researchers, journal editors, and peer-reviewers on the importance of prospective registration of protocols before the publication of reviews.

### Limitations

Our study has several limitations that should be considered when interpreting the findings: First, our analysis was based solely on protocols registered with PROSPERO. However, there are other platforms that researchers use for this purpose, e.g., systematic review protocols of animal studies used to be registered on the SYRCLE (Systematic Review Centre for Laboratory Animal Experimentation) website [34]. PROSPERO only accepts reviews with clear relevance to human health, introducing selection bias to our sample and potentially limiting generalizability. Additionally, platforms like the Open Science Framework (OSF) also offer services for pre-registering study protocols. Second, our method of determining the anticipated time to complete a systematic review is an underestimation of the ambition of some review authors. This is because administrators at PROSPERO4animals frequently suggest adjusting the anticipated completion date if it seems overly ambitious to complete the review. Third, we equated the completion of a review with its submission to a journal. This approach overlooks two key aspects: the possibility that a review might have been submitted to, and rejected by, other journals before finding a suitable one, and the time-consuming process of formatting a manuscript to adhere to a specific journal’s style guidelines [35]. These factors could result in an overestimation of the actual time taken to complete a systematic review. Fourth, PROSPERO permits registration at various stages of the review process, up to the point before data extraction begins. The registration stage likely affects the measured time to completion but could not be accounted for in our analysis. Despite these limitations, we believe our methodology remains relevant, as the true value of a systematic review lies not just in its completion but also in its availability to the public.

### Strengths

Our study provides a large-scale analysis of a substantial proportion of animal research systematic review protocols registered on PROSPERO, one of the main registries for such protocols.

## Conclusions

Only half of registered systematic review protocols of animal studies resulted in a publication. While this may suggest the presence of publication bias, other factors—such as limited time, lack of funding, or shifting priorities—may also contribute to non-publication. In addition, we provide concrete guidance on the expected timeframe for completing and publishing an animal systematic review. If they provide meaningful benefits, we also can encourage engaging in cross-country collaborations and incorporating diverse expertise since these practices do not increase the time required to complete reviews. Future research could identify the factors that influence the publication of animal systematic reviews. Understanding these factors could aid in promoting and improving the dissemination of this important research methodology.

Upon completing their protocol, authors submit it to the admin team for review. The admin team evaluates the content and decides whether to reject it, register it without revisions, or request revisions from the author (purple area). Once the initial version is registered, authors have the option to submit amendments to their protocol at any time, followed by admin team review (yellow area). Finally, upon completion of a systematic review, authors submit it to a journal which either rejects, refers for revisions, or publishes the study. Authors are asked to amend publication details to their respective protocols on PROSPERO (green area). Figure adjusted from [12].

## Supplementary Information

Supplementary Material 1.

## Data Availability

The datasets including the analysis code supporting the conclusions of this article are available in the Open Science Framework repository, at [https://osf.io/up5zr/](https://osf.io/up5zr). Specifically, the repository includes: 1) Grouped subject index terms: Manually classified topic categories based on PROSPERO subject index terms; 2) Screening and extraction dataset: Final dataset used for analysis, including publication status, timelines, and protocol characteristics; 3) Meta-data file: Describes each variable in the dataset, including definitions and coding criteria; 4) Analysis scripts: R scripts used for data cleaning, statistical analysis, and figure generation; 5) Web scraping scripts: Python code used to extract structured data from PROSPERO entries; 6) study protocol. For any questions regarding the data, meta-data, or analysis code, contact the corresponding author, BVI.
