# Supplementary material for: How long does it take to complete and publish a systematic review of animal studies?
Source: BMC Med Res Methodol. 2025 Oct 1;25:226. doi: 10.1186/s12874-025-02672-5 (PMC12487012; doi:10.1186/s12874-025-02672-5)
Supplement: Supplementary file 1 — Supplementary Material 1. [file 12874_2025_2672_MOESM1_ESM.docx]

How long does it take to complete and publish an animal systematic review?

Julia Victoria Bugajska^1^, Bernard Friedrich Hild^1^, David Brüschweiler^1^, Alexandra Bannach-Brown^3^, Kimberley Elaine Wever*^4^, Benjamin Victor Ineichen*^1,5^

**Affiliations:**

^1^ University of Zurich, Center for Reproducible Science, Zurich, Switzerland

^3^ QUEST Center for Responsible Research, Berlin Institute of Health at Charité-Universitätsmedizin Berlin, Berlin, Germany.

^4^ Radboud university medical center, Department of Anesthesiology, Pain and Palliative Medicine, Nijmegen, The Netherlands. Kim.wever@radboudumc.nl ORCID: https://orcid.org/0000-0003-3635-3660

^5^ University of Zurich, Clinical Neuroscience Center, Zurich, Switzerland

*Authors share senior authorship

**Correspondence to:**

Benjamin Ineichen, Center for Reproducible Science, University of Zurich, Switzerland. OrcID: 0000-0003-1362-4819. [benjamin.ineichen@uzh.ch](mailto:benjaminvictor.ineichen@uzh.ch)

# Supplementary data

**Supplementary Table 1:** Journals and timelines for publishing systematic reviews of animal studies.

| Journal Name | Count | Median time (days) | Minimum time (days) | Maximum time (days) |
| --- | --- | --- | --- | --- |
| bmc systematic reviews | 4 | 362.5 | 158 | 753 |
| systematic reviews | 5 | 324 | 235 | 444 |
| journal of oral and maxillofacial surgery | 3 | 302 | 68 | 336 |
| clinical nutrition espen | 3 | 280 | 200 | 577 |
| npj regenerative medicine | 3 | 226 | 175 | 231 |
| phytotherapy research | 6 | 225.5 | 157 | 317 |
| brazilian oral research | 3 | 217 | 194 | 497 |
| heliyon | 4 | 214.5 | 40 | 282 |
| phytomedicine | 5 | 202 | 117 | 308 |
| pain | 4 | 196 | 166 | 383 |
| environment international | 4 | 194.5 | 182 | 292 |
| bmj open science | 3 | 191 | 151 | 212 |
| journal of ethnopharmacology | 4 | 182.5 | 118 | 310 |
| the angle orthodontist | 3 | 182 | 180 | 254 |
| intensive care medicine experimental | 3 | 176 | 153 | 230 |
| oxidative medicine and cellular longevity | 8 | 160.5 | 82 | 278 |
| neuroscience and biobehavioral reviews | 16 | 156 | 52 | 435 |
| plos one | 16 | 154.5 | 88 | 255 |
| evidence based complementary and alternative medicine | 4 | 140 | 113 | 183 |
| osteoarthritis and cartilage | 4 | 140 | 118 | 251 |
| transboundary and emerging diseases | 3 | 136 | 88 | 196 |
| plos neglected tropical diseases | 3 | 126 | 91 | 221 |
| frontiers in immunology | 5 | 109 | 52 | 160 |
| biomed research international | 5 | 108 | 79 | 145 |
| molecular neurobiology | 4 | 108 | 89 | 313 |
| frontiers in bioengineering and biotechnology | 3 | 101 | 58 | 155 |
| frontiers in neuroscience | 3 | 95 | 54 | 96 |
| frontiers in pharmacology | 16 | 92.5 | 56 | 327 |
| tissue engineering | 4 | 92 | 70 | 521 |
| stem cell research and therapy | 9 | 87 | 51 | 261 |
| life sciences | 7 | 85 | 36 | 199 |
| frontiers in cardiovascular medicine | 3 | 83 | 80 | 395 |
| archives of oral biology | 6 | 81.5 | 50 | 136 |
| journal of functional foods | 3 | 73 | 73 | 79 |
| pharmacological research | 4 | 64 | 45 | 151 |
| antioxidants | 3 | 60 | 35 | 62 |
| medicine baltimore | 5 | 60 | 30 | 174 |
| environmental research and public health | 4 | 52.5 | 47 | 66 |
| nutrients | 11 | 41 | 12 | 82 |
| international journal of molecular sciences | 13 | 40 | 22 | 56 |
| animals | 3 | 35 | 30 | 67 |
| research society and development | 3 | 26 | 18 | 111 |
| materials | 3 | 21 | 20 | 28 |

Median, minimum, and maximum time from submission to publication for journals with ≥3 animal systematic reviews included in the analysis.

**Supplementary Table 2**: Topics of systematic review protocols

| Subject index terms grouping for >1 category | Count | percentage |
| --- | --- | --- |
| cardiovascular neuroscience | 44 | 3.22 |
| dental digestive | 34 | 2.49 |
| digestive neoplasms | 11 | 0.81 |
| metabolic urinary | 9 | 0.66 |
| neoplasms skin | 6 | 0.44 |
| digestive metabolic | 4 | 0.29 |
| neoplasms reproductive | 4 | 0.29 |
| digestive neuroscience | 3 | 0.22 |
| immunology neuroscience | 3 | 0.22 |
| metabolic musculoskeletal | 3 | 0.22 |
| cardiovascular immunology | 2 | 0.15 |
| metabolic neuroscience | 2 | 0.15 |
| neoplasms respiratory | 2 | 0.15 |
| cardiovascular digestive neuroscience | 1 | 0.07 |
| cardiovascular metabolic | 1 | 0.07 |
| cardiovascular metabolic neuroscience | 1 | 0.07 |
| cardiovascular metabolic skin | 1 | 0.07 |
| cardiovascular neoplasms | 1 | 0.07 |
| cardiovascular sensory | 1 | 0.07 |
| cardiovascular skin | 1 | 0.07 |
| digestive immunology | 1 | 0.07 |
| digestive infectiology | 1 | 0.07 |
| digestive urinary | 1 | 0.07 |
| immunology urinary | 1 | 0.07 |
| infectiology neuroscience | 1 | 0.07 |
| infectiology respiratory | 1 | 0.07 |
| metabolic musculoskeletal neuroscience | 1 | 0.07 |
| metabolic neuroscience reproductive | 1 | 0.07 |
| metabolic respiratory urinary | 1 | 0.07 |
| metabolic sensory | 1 | 0.07 |
| musculoskeletal neoplasms | 1 | 0.07 |
| musculoskeletal skin | 1 | 0.07 |
| neoplasms neuroscience | 1 | 0.07 |

Combinations of subject index terms assigned to protocols covering more than one biomedical research category, with corresponding frequencies and percentages.
